# Comparative genomics of ‘Xanthomonas cannabis’ reveals an emerging, diverse pathogen

**DOI:** 10.1099/mgen.0.001588

**Published:** 2025-11-28

**Authors:** Daniel J. E. McKnight, Lauren Clackson, Johanna Wong-Bajracharya, John Webster, Paul Worden, Fridtjof Snijders, Efenaide B. Okoh, Steven P. Djordjevic, Toni A. Chapman, Daniel R. Bogema

**Affiliations:** 1 NSW Department of Primary Industries and Regional Development, Elizabeth Macarthur Agricultural Institute, Woodbridge Rd, Menangle NSW 2568, Australia; 2 Australian Institute for Microbiology and Infection, University of Technology Sydney, Ultimo, NSW, Australia; 3 Hawkesbury Institute for the Environment, Western Sydney University, Penrith, NSW, Australia

**Keywords:** biosecurity, bioinformatics, genomic islands, pathogen, phylogenetics, type 3 secretion system (T3SS), *Xanthomonas*

## Abstract

We report the presence of the emerging plant pathogen ‘*Xanthomonas cannabis*’ in Australia through a comprehensive analysis of five historical isolates and all publicly available genomes of the species. Using comparative genomics, we characterized four isolates collected from *Zinnia* spp. and one from *Cucurbita pepo*. Our findings show that the *Zinnia* isolates form a distinct phylogroup with the pathotype strain of ‘*X. cannabis*’ pv. *zinniae*. This group possesses genes for the type 3 secretion system (T3SS) and effectors, a variety of genes unique within the species, and nine genomic islands associated with virulence and drug resistance. In contrast, the *C. pepo* isolate is genetically distinct and lacks the T3SS but contains its own genes unique within the species. Hypersensitivity response assays confirmed the pathogenic potential of all five isolates in black bean, eggplant, green bean, tomato, sunflower, zinnia and zucchini plants. These results highlight the genetic diversity and evolving threat of this pathogen in Australia, underscoring the critical need for ongoing biosecurity surveillance.

**Impact Statement**

This study provides the first confirmation of ‘*Xanthomonas cannabis*’ in Australia and characterizes the virulence factor repertoire and genetic diversity of five historic isolates. We identified a distinct group of Australian strains isolated from zinnia plants carrying unique virulence-associated genes and genomic islands not found in other members of the species. Crucially, our work demonstrates their pathogenic potential when infiltrated into the leaves of black bean, eggplant, green bean, tomato, sunflower, zinnia and zucchini plants.

In addition, this research represents the first comprehensive analysis of all publicly available ‘*X. cannabis*’ genomes, incorporating associated metadata to contextualize our findings. Furthermore, by compiling extensive historical phenotypic data and new genomic evidence, we provide the basis for the formal recognition of ‘*X. cannabis*’ as a species, resolving its long-standing unofficial taxonomic status. Our findings highlight an evolving threat and underscore the importance of ongoing biosecurity surveillance to protect Australian agricultural industries.

## Data Summary

All raw reads and assemblies are available from the GenBank database. Accession numbers for all genes are provided in Table S1 within the online Supplementary Material.

## Introduction

*Xanthomonas* is a genus of Gram-negative bacteria that is known for its extensive host range, capable of infecting over 400 plant species [1,2]. At present, there are 39 official species of *Xanthomonas* [3], many of which infect economically significant crops, including cereals, legumes, vegetables, fruits and ornamentals [1,2, 4]. Some of the most notable *Xanthomonas* diseases include citrus canker, bacterial leaf blight of rice and black rot of crucifers [5,7]. They cause significant economic losses worldwide by diminishing produce quality and causing crop loss [8].

The ability of *Xanthomonas* spp. to cause disease is mediated by their range of virulence factors, including the type 1 to 6 secretion systems (T1SS to T6SS). Each secretion system differs in structure but generally consists of a macromolecular tube that translocates protein effectors, toxins or adhesins out of the cell. Of these, the type 3 secretion system (T3SS) and its type 3 effectors (T3Es) are considered a major virulence determinant in *Xanthomonas* species [9,10].

The T3SS is a needle-like multiprotein complex that translocates proteins across the bacterial cell membrane into the cytoplasm of a host [11]. It translocates T3Es into the host cell, manipulating plant cellular pathways to favour the pathogen [12]. This includes interfering with immune response, molecular signalling, photosynthesis, gene expression and cytoskeleton formation [13]. The T3SS is encoded by the hypersensitivity reaction and pathogenicity (*hrp*) genes, which are non-essential in media-based growth but are required for hypersensitivity reactions in plants. The most downstream proteins in the *hrp* regulatory cascade are HrpG and HrpX, where HrpG regulates the expression of HrpX, a transcriptional activator, that ultimately leads to the expression of T3SS and T3E [9,14]. However, HrpG and HrpX are part of a larger regulatory network, interacting with other signalling molecules and transcriptional regulators. They also control diverse cellular functions beyond the *hrp* T3SS, including the expression of cell wall-degrading enzymes, genes involved in chemotaxis and motility, and preparation for the import and metabolism of plant-derived compounds [15,16].

In *Xanthomonas* species, the *hrpX* and *hrpG* genes are often located in or near the *hrp* gene cluster [17,18]. When mutant strains lack either *hrpX* or *hrpG*, they become non-pathogenic, as they do not express the *hrp* gene cluster [19,21]. This regulatory process involves the binding of HrpX to a conserved cis element known as a plant-induced promoter (PIP) box. The PIP box also requires a properly spaced −10 promoter motif, resulting in the sequence TTCGBN_15_-TTCGB-N_30−32_-TYNNNT [17]. When HrpX, HrpG and the PIP box with its −10 promoter motif are present, the *hrp* T3SS gene cluster can be expressed.

Beyond the T3SS, secretion systems 1–6 (T1SS–T6SS) also contribute to the adaptation and virulence of *Xanthomonas* species [9]. These systems secrete a variety of effectors, including toxins, metal scavenger proteins, adhesins, degradative enzymes and genetic material [22,24]. Depending on the secretion system, the effectors are translocated into the extracellular space, or directly into the cytoplasm of target plant hosts or competing bacteria [24]. Effectors have a range of functions, including biofilm formation, suppression of host immune response, host cell degradation, nutrient acquisition, adhesion, toxin secretion, conjugation of genetic material and stress response [9,25, 26]. This allows them to outcompete bacteria in the immediate environment, infiltrate and colonize hosts and increase their biological fitness [9,24]. The role of these systems in *Xanthomonas* spp. is reviewed in greater detail in Alvarez-Martinez *et al*. [9].

Other common virulence-associated factors found in *Xanthomonas* spp. include lipopolysaccharides (LPSs), which aid in cell adhesion, biofilm formation, reduced permeability of outer membrane to increase stress tolerance and suppression of hypersensitivity reactions in hosts [27,29]. Conversely, LPS can also elicit pathogen‐associated molecular pattern defence‐related responses in plant hosts [30]. The LPS gene cluster is located between electron transport flavoprotein subunit A (*etfA*) and cystathionine gamma-lyase (*metB*), two highly conserved housekeeping genes [31]. Therefore, instead of screening for all LPS genes, the presence of the *metB* and *etfA* can be used as reliable markers for the LPS gene cluster.

Similar in function, *Xanthomonas* spp. are well known for their exopolysaccharide (EPS), xanthan, that causes the mucoidal appearance of their colonies. EPS aids in cell adhesion, stress tolerance and biofilm formation [32]. Therefore, a thorough investigation of potentially pathogenic *Xanthomonas* species requires analysis of secretion systems and their associated effectors and regulators, as well as the presence of EPS and LPS.

Genomic islands (GIs) are discrete genetic regions acquired through horizontal gene transfer that often contain clusters of genes related to specialized functions, such as virulence, antibiotic and metal resistance and metabolic adaptations [33]. Using modern tools like IslandCompare and Mauve, GI can be rapidly identified and analysed to determine their distribution between isolates. The acquisition of GI can lead to significant changes in the phenotype of the organism, including the ability to infect new hosts or resist certain antibiotics. Therefore, tracking the presence and distribution of GI can provide insight into the adaptive evolution of the species.

Since the *Xanthomonas* genus was first proposed in the early 20th century, taxonomic classification of species and subspecies groups has been debated. This is due to *Xanthomonas* spp. originally being identified by phenotypic analysis or host range. However, as molecular biology and sequencing technologies have developed, our ability to distinguish between species has greatly increased. As a result, many original *Xanthomonas* spp. have been subdivided into many new species, while others have been amalgamated [34]. Additionally, pathovars have been shuffled between *Xanthomonas* species based on DNA sequence data [35]. Modern advances in sequencing technology now provide high discriminatory power and techniques like phylogenomics and genomic relatedness indices [e.g. average nucleotide identity (ANI)] that allow researchers to determine genetic relationships between highly related isolates. These techniques have greatly aided in species delineation, as they provide a more accurate framework for understanding genetic relationships, though taxonomy itself remains subject to revision as new data emerges.

In 1947, Watanabe reported a bacterial disease in hemp and identified the causal agent as ‘*Pseudomonas cannabis*’ [36]. Then, in 1955, Okabe and Goto suggested renaming this disease to ‘*Xanthomonas cannabis*’, but they did not deposit a type strain [37]. Later, in 1978, Severin reported a similar hemp disease in Romania, which was referred to as *Xanthomonas campestris* pv. *cannabis* [38]. In 2009, Parkinson *et al.* utilized *gyrB* sequence analysis to determine that *X. campestris* pv. *cannabis* belongs in its own species-level clade [39]. However, the following year, Bull *et al*. released a comprehensive list of names of plant pathogenic bacteria, referring to it as *X. campestris* pv. *cannabis*, as suggested by Severin [40]. In 2014, Netsu *et al*. presented evidence supporting the findings of Parkinson *et al.*, further reinforcing the classification of *X. campestris* pv. *cannabis* as a distinct species [41]. The following year, Jacobs *et al*. proposed that two representative strains of *X. campestris* pv. *cannabis* should be renamed to ‘*X. cannabis*’, with an isolate collected by Severin (NCPPB 2877) as the type strain [17,38]. They reported that in pathogenicity trials, the isolates were capable of infecting cannabis, barley, tobacco and capsicum. Despite clear genomic evidence supporting its classification as a distinct species, it remains unofficially recognized as it did not fulfil the criteria for official species description [42]. Since its first report, ‘*X. cannabis*’ has been collected from several plant genera, including *Abelmoschus*, *Cannabis*, *Phaseolus*, *Solanum* and *Zinnia*. Within the ‘*X. cannabis*’ species, there are currently four proposed pathovars: pv. *cannabis*, pv. *esculenti*, pv. *phaseoli* and pv. *zinniae* [17,43].

*X. campestris* pv. *zinniae* (a.k.a. *Xanthomonas nigromaculans* pv. *zinniae*) causes necrotic lesions in flowers and leaves of *Zinnia* spp. and is a threat to *Zinnia* cultivation. Additionally, previous studies have identified necrotic leaf spots in tomatoes on exposure to pv. *zinniae* [44]. The *zinniae* pathovar was first proposed in 1948 as *X. campestris* pv. *zinniae* after bacterial leaf spot was observed on *Zinnia* plants [45]. Presence in Australia was first reported in 1971 as *X. nigromaculans* f. sp. *zinniae* on *Zinnia* hosts from Armidale, Australia [46]. Recent genomic analysis of the isolate NCPPB 2439, the current pathotype of pv. *zinniae*, demonstrated high similarity with isolates of ‘*X. cannabis*’, and the pathovar has now been proposed to be reclassified [43].

Despite its pathogenic nature, pathovar *zinniae* possesses the ability to degrade the toxin cercosporin into a non-toxic metabolite, xanosporic acid. Cercosporin, produced by various species in the fungal genus *Cercospora*, is a non-host-specific toxin that threatens a wide array of plant species. A study that screened 244 bacterial isolates from 12 genera identified *X. campestris* pv. *zinniae* and *X. campestris* pv. *pruni* as the most efficient at degrading cercosporin [47]. Significantly, pathovar *zinniae* can catabolize all cercosporin within 60 h into metabolites proven to be non-toxic to tobacco leaves [47,48]. Further work from these researchers identified the specific oxidoreductase enzyme that mediates this degradation and a transcriptional regulator required for its function [49]. Investigations of these genes in pathovar *zinniae* could provide information relevant to developing *Cercospora*-resistant plants.

During routine surveillance from 1977 to 1984 across New South Wales (NSW) and Queensland (QLD) in Australia, four ‘*X. cannabis*’ isolates were collected from *Zinnia* sp. and one from *Cucurbita pepo* (zucchini). We analysed their intraspecific relationship with other members of the ‘*X. cannabis*’ species and used bioinformatic techniques to identify the presence of virulence-associated factors commonly found in pathogenic *Xanthomonas* species. We also performed hypersensitivity response (HR) assays to determine their pathogenic potential.

## Methods

### Isolation, propagation, sequencing and assembly

Bacterial samples were isolated from diseased *Zinnia* sp. (DAR 41374, DAR 82727, DAR 82752 and DAR 82756) and *C. pepo* (DAR 41331) between 1977 and 1984 in NSW and QLD, Australia. DAR 41331 was isolated from angular leaf spot, while DAR 41374 and DAR 82756 were collected from leaf spot. However, this metadata is unfortunately missing for DAR 82727 and DAR 82752. They were collected as part of routine biosecurity surveillance by the NSW Department of Primary Industries and stored in the NSW Plant Pathology and Mycology Herbarium. They were initially identified as *Xanthomonas* using phenotypic techniques and were believed to belong to the *Xanthomonas cucurbitae* and *X. campestris* species. The preserved cultures were lyophilized, sealed under vacuum in glass ampoules and stored at 4 °C. The preserved specimens were recovered onto yeast dextrose carbonate solid agar and incubated for 48 h at 25 °C. The DNA extraction, sequencing and assembly of isolates were conducted according to McKnight *et al*. [50]. In summary, the bacterial cultures were grown from lyophilized samples, genomic DNA was extracted using a Nanobind CBB Big DNA Kit (Circulomics), and the DNA was sequenced on a PromethION R9.4 flow cell. Raw reads were assembled using Flye v2.9-b1768, Miniasm v0.3-r179 with Minipolish v0.1.2, Raven v1.8.1 and Necat v0.0.1_update20200803 [51,52]. Draft assemblies were combined with Trycycler and polished with long reads using Medaka v1.7.1. They were further polished using Illumina short reads, which were generated from prior unpublished work, using Polypolish v0.5.0 and POLCA from MaSuRCA v4.0.9 [53,55].

### Species determination and intraspecific analysis

The taxonomic classification of our isolates was determined by comparing their genome sequences against those of all currently recognized *Xanthomonas* type strains using FastANI v1.32 [56]. The resulting ANI values were used as the basis for classification, with isolates exhibiting an ANI value exceeding 95% being considered as belonging to the corresponding *Xanthomonas* species [57]. To find all publicly available ‘*X. cannabis*’ genomes, including those that may have been misidentified, we downloaded all isolates listed under the *Xanthomonas* genus on the National Center for Biotechnology Information (NCBI) database using NCBI Genome Download v0.3.1 [58]. FastANI v1.32 [56] was used to compare these NCBI genomes with a confirmed ‘*X. cannabis*’ strain (NCPPB 2877 GCF_000802365.1) as a reference and calculate the ANI. Genomes exhibiting an ANI value exceeding 95% were retained as members of the ‘*X. cannabis*’ species for subsequent analysis. The intraspecific relationships amongst these isolates were determined via pairwise ANI calculations using FastANI v1.32 [56] and were visualized using iTOL [59].

### Type strain UBCG phylogeny

Our five *Xanthomonas* isolates and all *Xanthomonas* type strains were used to generate a 92-gene multilocus phylogeny with *Pseudoxanthomonas suwonensis* strain 11-1 (GCF_000185965.1) as the outgroup. UBCG v3 [60] was used with default settings to extract and concatenate the 92 core genes which were aligned using MAFFT v7.310 [61]. The *Xanthomonas pisi* type strain was replaced with the NCBI reference genome (GCF_002940045.1) because the type strain genome is incomplete and missing many core genes. A maximum likelihood phylogeny was constructed using IQ-TREE v2.1.4 with the GTR+F+R4 model and 100 non-parametric bootstrap replicates [62]. It was then outgroup rooted and visualized using iTOL [59].

### ‘*X. cannabis*’ core gene phylogeny

A core gene phylogeny including all ‘*X. cannabis*’ isolates from the NSW Plant Pathology and Mycology Herbarium and NCBI was created using Core_gene_phylo v1.0.0 [63] as per Webster *et al*. [64]. The core gene phylogeny was combined with metadata collected from multiple genome databases and associated literature. This combined phylogeny and metadata was then visualized and midpoint rooted in iTOL [59].

### Core SNP tree

A core genome SNP phylogeny of all ‘*X. cannabis*’ isolates found in Australia was generated using Snippy v4.6.0 [65]. DAR 82752 was used as the reference as it has the longest genome of the isolates. The core genome SNP alignment output from Snippy was used to generate a phylogeny using IQ-TREE v2.1.4 [62]. The TVM+F+ ASC model was used and 100 bootstrap replicates were generated. The phylogeny was midpoint rooted and visualized in iTOL.

### Virulence-associated factors

Virulence-associated factors were identified using a reference database of 123 previously characterized *Xanthomonas* genes from Gétaz *et al.* [66] combined with the T3E from The *Xanthomonas* Resource [67]. Genes from Gétaz *et al*. that encode for T3SS flagellar genes were not included in the analysis. This database contained genes for extracellular polysaccharides (EPS), LPS, T3SS, T3E, type 4 secretion system (T4SS) and type 6 secretion system (T6SS). The genes *estA*, *fhaB*, *hlyB*, *pctB*, *raxB*, *xadA*, *xcs* and *xps* were used to signal the presence of the T1SS, type 2 secretion system (T2SS) and type 1 secretion system (T5SS) [9]. Cercosporin degradation ability was assessed by searching for the associated oxidoreductase and transcriptional regulator genes (*oxR*) [49]. Gum genes (*gumB* to *gum*N) were extracted from a *Xanthomonas oryzae* pv. *oryzae* genome (AE013598.1). All gene accession numbers can be found in Table S1 (available in the online Supplementary Material). Bakta v1.5.1 [68] was used to annotate the genomes, and Diamond v2.0.15 [69] with no limit on maximum target sequences was used for genome searching using blastx. Positive hits were counted if they were ≥70% length similarity, ≥70% alignment similarity and an e-value ≤1e^−10^. Alignment length similarity was ensured by setting Diamond’s --subject-cover parameter to 70, while alignment similarity and e-value were manually filtered in Microsoft Excel. Presence/absence data was visualized using R v4.3.0 [70] with the R package Pheatmap v1.0.12 [71]. The positions of *hrpX* and *hrpG* were found through manual inspection of Bakta annotations as well as with blast using reference sequences from previous studies [14,18].

### Pangenome and GI analysis

To determine what genes are unique to our isolates and NCPPB 2439 compared to other members of the species, the NCBI GenBank annotations of all ‘*X. cannabis*’ genomes were downloaded. These GenBank files were processed by Roary v3.13.0 [72] using default settings and visualized using RoaryPlots. The pangenome reference file was then processed using Scoary v1.6.16 [73] to identify genes unique within the species. Coding sequences that were unique to each group were analysed by EggNOG-Mapper v2.1.12 [74] to determine their function.

To further confirm the uniqueness of these genes to each group, we performed additional analysis. We downloaded the protein sequences of all members of the ‘*X. cannabis*’ species from the NCBI and searched each genome for the putative unique genes using Diamond v2.0.15 [69] with blastp. This search employed significantly broader similarity parameters (≥70% length similarity, ≥70% alignment similarity and an e-value ≤1e^−10^) compared to Roary’s default 95% blastp similarity threshold [72]. All sequences identified by blastp in other members of the ‘*X. cannabis*’ species were removed from the final analysis. Additional manual analysis of some sequences was performed using Geneious Prime 2023.0.4 (Dotmatics, Boston, USA). Data was visualized in R v4.3.0 [70] using the ggplot2 package [75].

The EMBL files from Bakta for the zinnia phylogroup were uploaded to Island Compare [76] to search for GIs. The output was analysed using Geneious Prime 2023.0.4 to determine their function. Manual inspection of each GI identified by IslandCompare was performed using Mauve v20150226 [77]. PHASTEST [78] was used to identify and annotate the presence of phage genes within the GI [1]. GI and genomes were visualized using BRICK v0.4.0 [79] with blast+ v2.14.0 [80] using a sequence length and similarity threshold ≥70% and an e-value ≤1e^−10^. DAR 82752 was used as the reference as it had the largest genome and was the only isolate that contained all 9 GIs.

### Hypersensitivity response assay

A pilot study was conducted based on the techniques used in the second trial of Kałużna *et al*. [81] to determine the best inoculation method for a full-scale HR assay. Steridium PLEDT-RH1250 plant growth chambers were used to grow 30 eggplant and 30 tomato plants at 60% humidity with a 20 °C 16-h day cycle and 8-h 16 °C night cycle. Isolates DAR 41331 and DAR 82756 were individually inoculated into sterile water to an approximate concentration of 1×10^8^ c.f.u. ml^−1^. The isolates were each applied to 15 eggplants and 15 tomato plants using three inoculation techniques, making 5 replicates for each of the 6 groups. The inoculation methods include syringe infiltration on the abaxial leaf surface, leaf infiltration with forceps wrapped in sterile cotton soaked in the bacterial suspension and rubbing the leaf surface with a mixture of bacterial suspension and carborundum powder. The syringe and forceps methods were applied six times to the three newest leaves on the eggplants and four times on each of the four newest leaves on the tomatoes due to their small leaf size. Results for the syringe method were the most promising and were consequently used for the full-scale HR trial.

Each isolate and a sterile distilled water control were infiltrated into the leaves of host plants with a needleless syringe. Six replicate plants were used for each of the following: *Solanum melongena* ‘Monarca’, *Solanum lycopersicum* ‘Colibri’, *Helianthus annuus* ‘EOS F1’, *Zinnia elegans* ‘Purple Prince’ and *C. pepo* ‘Apollonia’. Due to poor germination, a reduced number of replicates were used for *Phaseolus vulgaris* ‘Strike’ (*n*=2) and ‘Black Turtle’ (*n*=5). This required a total of 222 plants to perform the HR trial, and all assays were run for a minimum of 2 weeks.

From each plant, the four newest leaves were inoculated a maximum of six times, with smaller leaves receiving fewer inoculations based on their size. To eliminate any risk of cross-contamination, plants in each group were individually infected with fresh gloves, kept well spaced from each group and watered in individual saucers.

To confirm that the inoculated isolate was the causal agent of the observed symptoms, they were extracted and examined using MALDI-TOF MS with a Biotyper Microflex LT (Bruker Daltonics). When symptoms other than typical bacterial leaf spot were observed, an additional extraction was performed. This involved selecting four distinct disease symptoms from four different plants. These symptomatic leaf tissues were extracted and submerged in 400 µl of sterile water. The leaves were then macerated using sterilized scissors and left to soak for 15 min. Then, 100 µl of the mixture was plated, then streaked onto nutrient agar (NA) and incubated at 21 °C for 48 h. A single colony was transferred to an MBT Biotarget plate and overlayed with 1 µl of 70% formic acid. Once dry, 1 µl of *α*-cyano-4-hydroxycinnamic acid was then added and left to dry, before inserting the target plate into the Biotyper Microflex LT. Each run was performed using the settings outlined in Nellessen and Nehl [82] with a ratio of total-laser-shots to laser-shots-per-raster-spot of 5,000 :250. Spectra were analysed using MBT Compass software (v5.1.3) against both the MBT Compass reference library and a custom database containing spectra of all ‘*X. cannabis*’ isolates used in the HR trial. Identification was based on log score thresholds derived from the Bruker MALDI Biotyper Clinical Application manual: scores ≥2.0 signified secure species-level identification and scores ≥1.7 indicated reliable genus-level identification. The MALDI-TOF MS spectra file of each isolate has been uploaded to Figshare to aid in future identification: https://doi.org/10.6084/m9.figshare.29555630.v1.

## Results

**Fig. 1.**
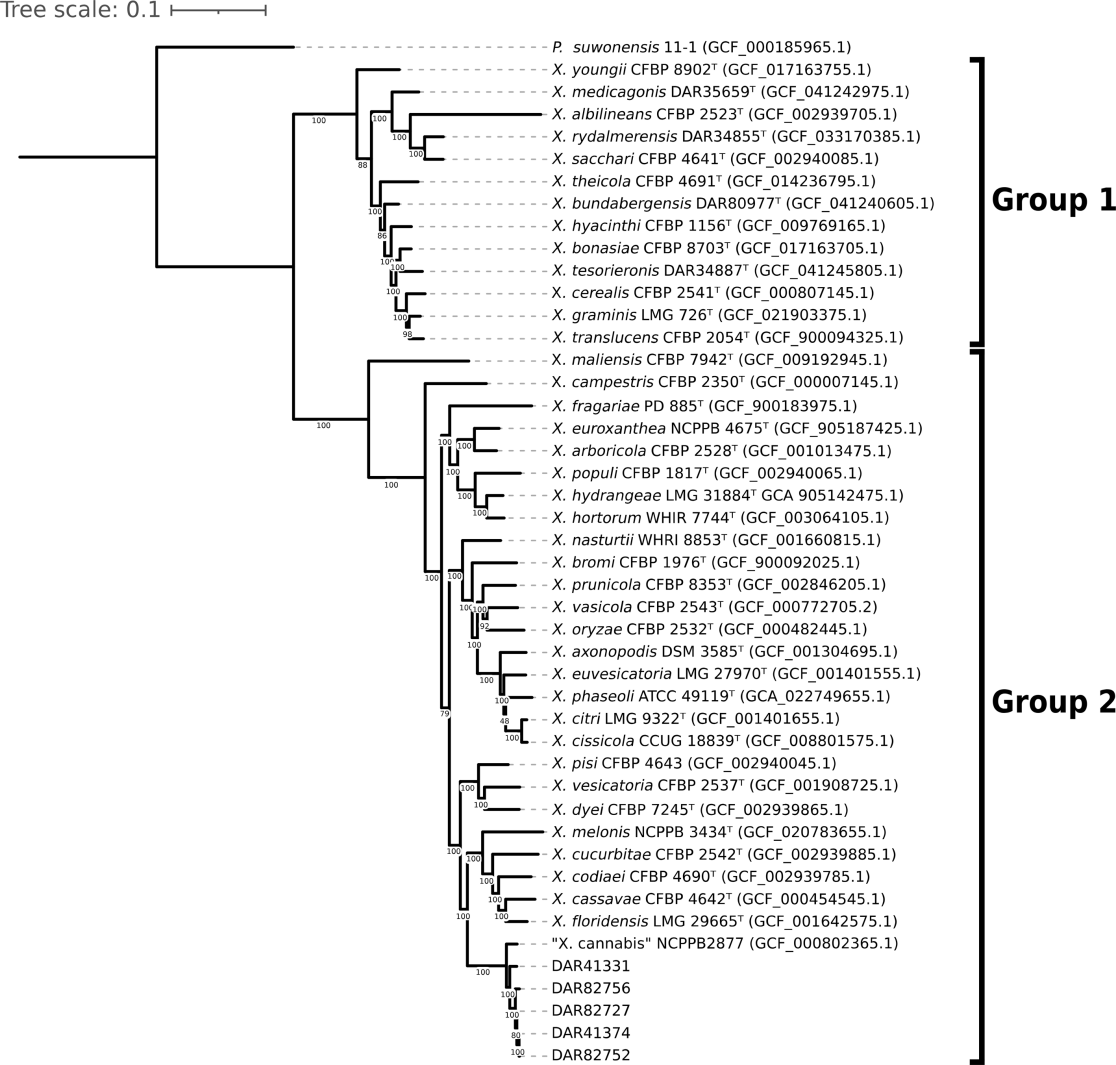
Outgroup rooted maximum-likelihood 92 gene multilocus (UBCG) phylogeny of all *Xanthomonas* type strains, Australian ‘*X. cannabis*’ isolates and *P. suwonensis* as the outgroup. Type strains are listed as species name, followed by strain name and RefSeq database number in parentheses. Numbered branches indicate bootstrap values, with 100 bootstrap replicates used to generate the tree.

To determine the species of our isolates (DAR 41331, DAR 41374, DAR 82727, DAR 82752 and DAR 82756), we generated a maximum likelihood core gene tree to compare them to all *Xanthomonas* type strains (Fig. 1). Our isolates grouped discretely with the ‘*X. cannabis*’ pv. *cannabis* pathotype strain (NCPPB 2877, GCF_000802365.1) in a monophyletic clade. This clade is within the *Xanthomonas* group 2, as described by Young *et al*. [83].

For further evidence to confirm their species, we compared our isolates to all *Xanthomonas* type strains using ANI. When compared to a well-characterized *‘X. cannabis*’ strain (NCPPB 2877), they produced values >96.5%. The next most closely related species were *Xanthomonas nasturtii* and *Xanthomonas codiaei* with ANI values less than 89.7% and 88.5%, respectively. All raw ANI values can be found in Table S2.

To investigate the intraspecific relationships between all members of the ‘*X. cannabis*’ species, NCPPB 2877 was used as a reference and compared to the 2,770 genomes labelled as *Xanthomonas* in the NCBI database. A total of 15 genomes exhibited ANI values above the species delineation threshold, ranging from 96.37% to 99.19%. Out of these 15 isolates, 11 were designated as ‘*X. cannabis*’ in the database, while 2 were labelled as *Xanthomonas* sp. (CFBP 7912 and CFBP 7698) and the remaining 2 were identified as *X. campestris* pv. *zinniae* and pv. *esculenti* (NCPPB 2439 and NCPPB 2190). The two isolates denoted as *Xanthomonas* sp. were utilized in the analysis of two scientific papers concerning the evolutionary aspects of T3SS in *Xanthomonas* species, where they were correctly referred to as ‘*X. cannabis*’ [84,85]. Likewise, the two isolates labelled as *X. campestris* were mentioned in a paper that suggested their inclusion within the ‘*X. cannabis*’ classification [43]. Therefore, despite their taxonomic metadata in the NCBI database, all four isolates are known members of ‘*X. cannabis*’. All 15 NCBI genomes identified as ‘*X. cannabis*’ using ANI were used in subsequent analyses.

**Fig. 2.**
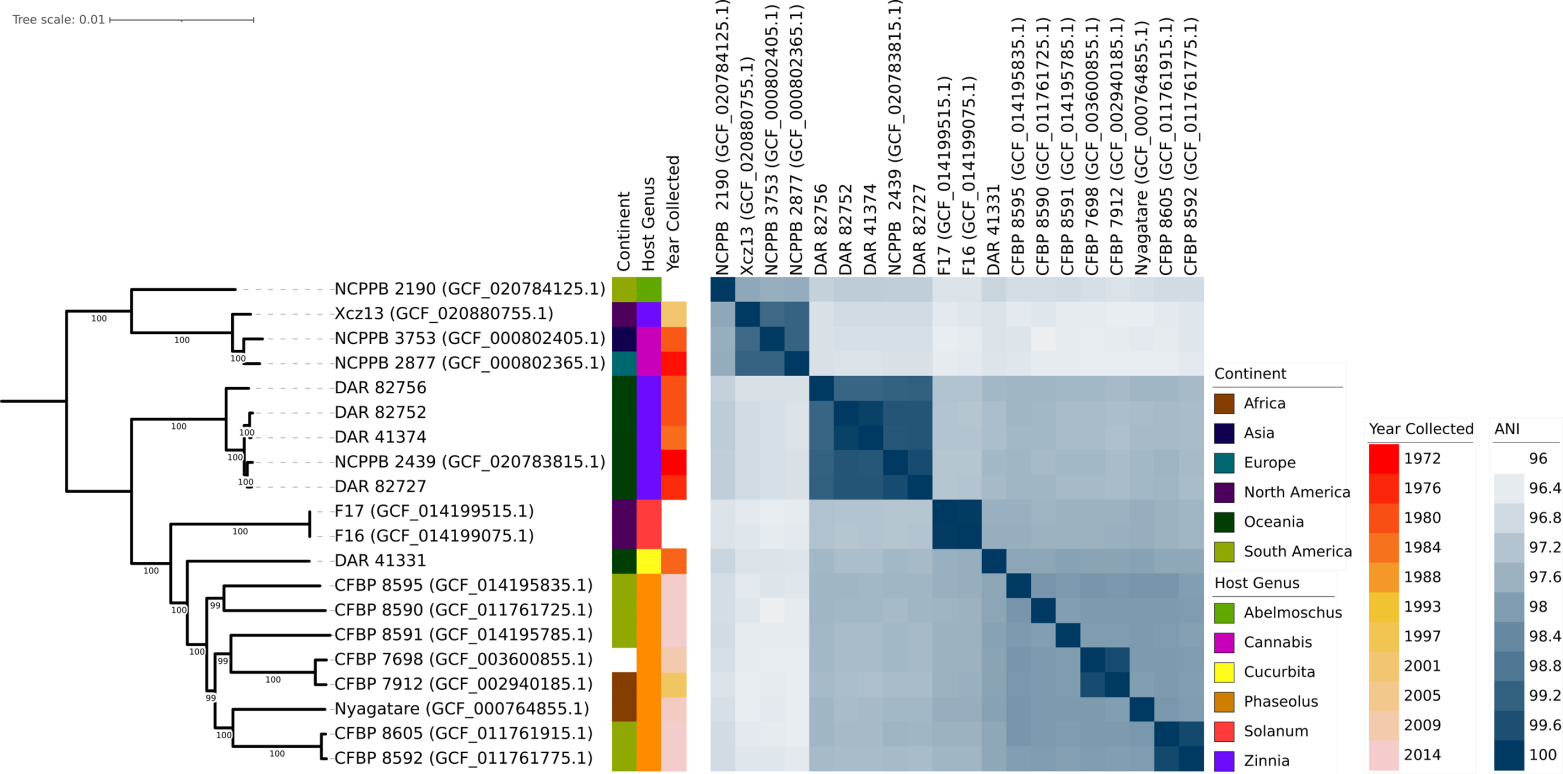
Midpoint-rooted core gene phylogeny of all ‘*X. cannabis*’ from the NSW Plant Pathology and Mycology Herbarium and NCBI. NCBI isolates are written as strain name followed by RefSeq database number in parentheses. White gaps in metadata signify that the metadata is not publicly available for a given isolate. All metadata can be found in Table S3 of the supplementary data.

We conducted a comparative analysis of our isolates with the 15 ‘*X. cannabis*’ NCBI genomes using ANI and core gene phylogeny. The ANI data was visualized as a heatmap, and the phylogeny was coupled with the metadata of each isolate including location, year collected and plant it was collected from (Fig. 2). Both techniques made it clear that the species has diverged into two distinct groups. One clade comprises NCPPB 2877, NCPPB 3753, Xcz13 and NCPPB 2190, which are significantly divergent from other members of the species. The second group includes the remaining 11 strains, including our isolates. This clear phylogenetic division and ANI values only 0.37% above the species delineation threshold indicate a very divergent taxonomic group.

The analyses also revealed a tightly clustered monophyletic clade comprising four of our isolates (DAR 41374, DAR 82727, DAR 82752 and DAR 82756) and NCPPB 2439, the ‘*X. cannabis*’ pv. *zinniae* pathotype strain. They present very high pairwise ANI values ranging from 99.15% to 99.86% and were collected from *Zinnia* sp. in Australia from 1972 to 1984. These five isolates will herein be referred to as the zinnia phylogroup.

Additionally, there were three pairs of isolates – CFBP 8592 and CFBP 8605, CFBP 7912 and CFBP 7698 and F16 and F17 – that exhibit high ANI and short phylogenetic branch lengths. F16 and F17 were found to have an ANI value of 100, indicating that they are clonal. The phylogeny also highlights eight isolates collected from bean plants including the ‘*X. cannabis*’ pv. *phaseolis* pathotype strain. They were collected within 14 years of each other in Africa, South America and one with unknown origin. Despite these similarities in collection time and plant source, they are genetically distant with two closer pairs and four single isolates with long branch lengths and lower ANI values.

Pangenome analysis of all members of the ‘*X. cannabis*’ species showed that they contain a total of 8,536 homologous gene groups, consisting of 3,050 core genes (present in 99–100% of isolates), 154 soft core genes (95–99%), 1,743 shell genes (15–95%) and 3,589 cloud genes (0–15%). NCBI genomes ranged from 4,706,139 to 5,098,330 bp in length and had between 10 and 260 contigs. Our isolates had fully circularized chromosomal DNA with no plasmids. Four of them ranged from 5,020,624 to 5,044,257 bp in length, while DAR 41331 was shorter with a length of 4,797,729 bp. In Fig. 2, our isolates with longer genomes exhibit close relatedness and form a distinct group, while DAR 41331 groups discretely from all other members of the ‘*X. cannabis*’ species.

**Fig. 3.**
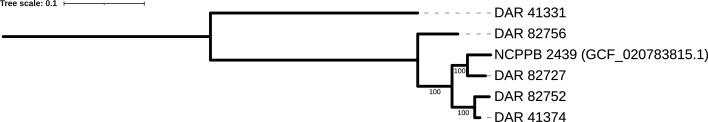
Midpoint-rooted core genome SNP phylogeny of all Australian members of the ‘*X. cannabis*’ species.

To better understand the genetic relationship between our isolates and the additional Australian isolate obtained from NCBI (NCPPB 2439), we constructed a core genome SNP phylogeny (Fig. 3). The pairwise SNP distance between these isolates ranged from 5665 in DAR 41374 to 84,666 in DAR 41331. The substantially larger number of SNPs and discrete clustering in the phylogeny further shows that DAR 41331 is distantly related to the other Australian isolates. Conversely, these findings highlight the high genetic similarity of four of our isolates to the pathotype strain NCPPB 2439, with as few as 5665 SNPs over a 5 Mbp genome.

**Fig. 4.**
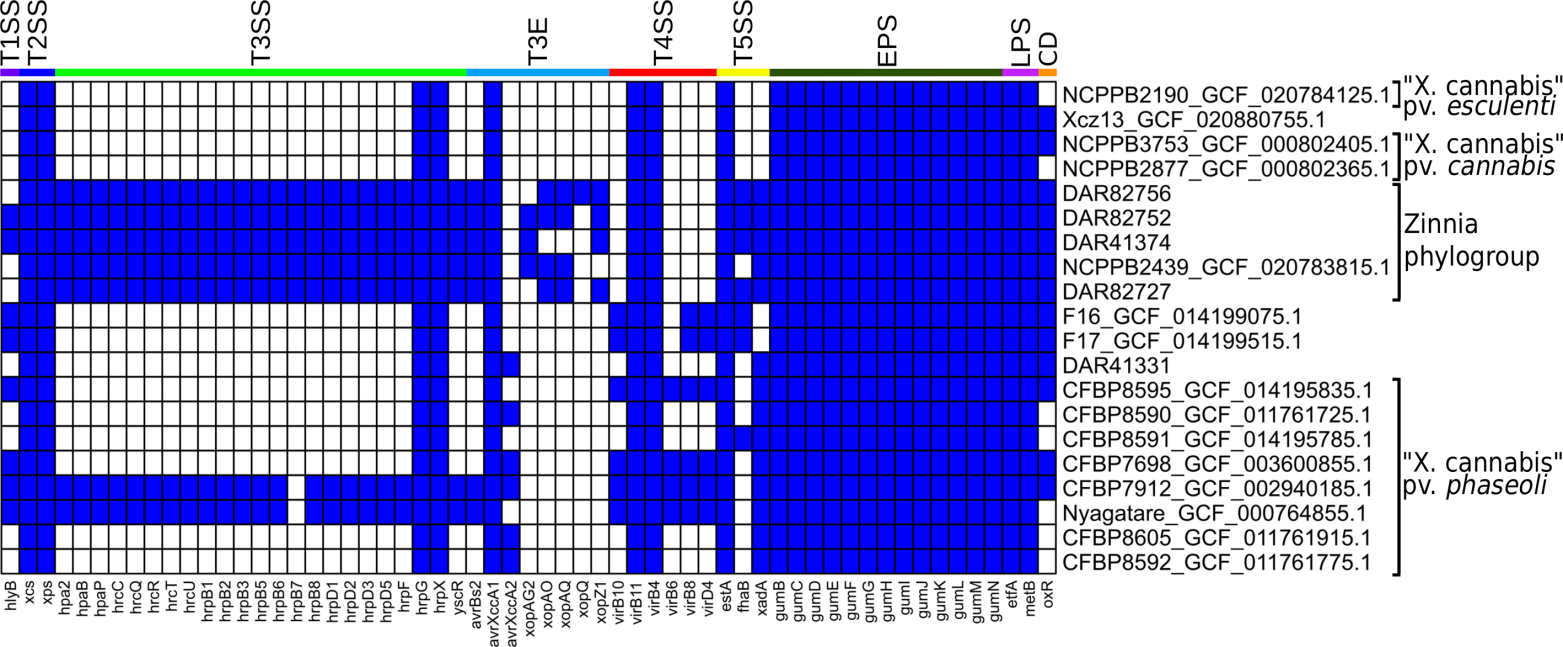
blast results of all ‘*X. cannabis*’ isolates with ≥70% length similarity, ≥70% alignment similarity and an e-value less ≤1e^−10^. Blue and white represent the presence and absence of a homologous gene, respectively. Larger labels on the coloured bands represent which virulence system each gene belongs to. CD, cercosporin degradation. NCBI isolates are written as strain name followed by RefSeq database number.

We investigated the genomes of all ‘*X. cannabis*’ isolates using blast to search for known virulence-associated factors (Fig. 4). All ‘*X. cannabis*’ isolates contained the following genes: both the *xcs* T2SS and *xps* T2SS, *hrpG* and *hrpX*, the T3E *avrXccA1*, *virB4* and *virB11* from the T4SS, *estA* from the T5SS, the entire gum operon and the LPS marker genes *etfA* and *metB*. Presence/absence analysis shows that the majority of ‘*X. cannabis*’ genomes lack the T3SS gene cluster. The exceptions are the zinnia phylogroup, Nyagatare and CFBP 7912 which possess 22–23 T3SS genes. Excluding avirulence genes (avrBs2, avrXccA1 and avrXccA2), the only genomes that contained T3E were the pathovar *zinniae* pathotype strain NCPPB 2439 and four of our Australian isolates. A polyphyletic group of isolates (F16, F17, CFBP 7698, CFBP 8595, CFBP 7912 and Nyagatare) was found to contain 3–4 T4SS genes that are absent from all other strains. The T5SS-associated gene *estA* was detected in all members of the ‘*X. cannabis*’ species, while two other T5SS-related genes, *fhaB* and *xadA*, were identified in 7 and 14 genomes, respectively. Only one of the three T1SS-associated genes (*hlyB*) was detected, which was found in CFBP 7698, CFBP 7912, CFBP 8595, F16, F17, Nyagatare and two members of zinnia phylogroup (DAR 82752 and DAR 41374). None of the 19 T6SS genes in our reference database were detected in any members of the ‘*X. cannabis*’ species. The presence of the oxidoreductase and transcriptional regulator genes (DQ087176) required for cercosporin degradation was found in 13 of the 20 ‘*X. cannabis*’ isolates, including all 5 isolates in the zinnia phylogroup.

Furthering our investigation into the virulence-associated genes of the zinnia phylogroup, we determined the positioning of major pathogenicity regulators. The genes *hrpX* and *hrpG*, regulators of the *hrp* regulatory cascade, were not positioned near the *hrp* cluster. Instead, they were located next to *radA,* ~1 Mbp downstream. This was checked against the ‘*X. cannabis*’ strain NCPPB 2877 and found similar positioning, with *radA*, *hrpX* and *hrpG* next to each other, while the *hrp* cluster could not be located, consistent with prior reports [17]. The T3SS genes in the zinnia phylogroup are clustered in approximately the same ~37,000 bp region of each genome. However, with NCPPB 2439 being an incomplete genome, the T3SS genes were positioned at the end of contig 13. PIP boxes with properly spaced promoter regions were also detected in the genomes of all five isolates. NCPPB 2439 had seven PIP boxes, DAR 82756 had five and the other three isolates had six. PIP boxes were found within or near the *hrp* cluster for all five isolates excluding NCPPB 2439.

The LPS gene cluster was manually investigated in the zinnia phylogroup, guided by the coordinates of the flanking *etfA* and *metB* genes. They were found to contain 15–18 genes, many of which were LPS-associated genes like glycosyltransferase and methyltransferase. Interestingly, the LPS cluster in DAR 82752 and NCPPB 2439 was 100% identical down to nucleotide level, while DAR 41374 and DAR 82756 were 99% identical, with small genetic variability. DAR 82727 was the most unique amongst them, containing genes not observed in the others like a membrane-associated protein, three oxidoreductase proteins, UbiA family prenyltransferase and flippase-like domain-containing protein.

**Fig. 5.**
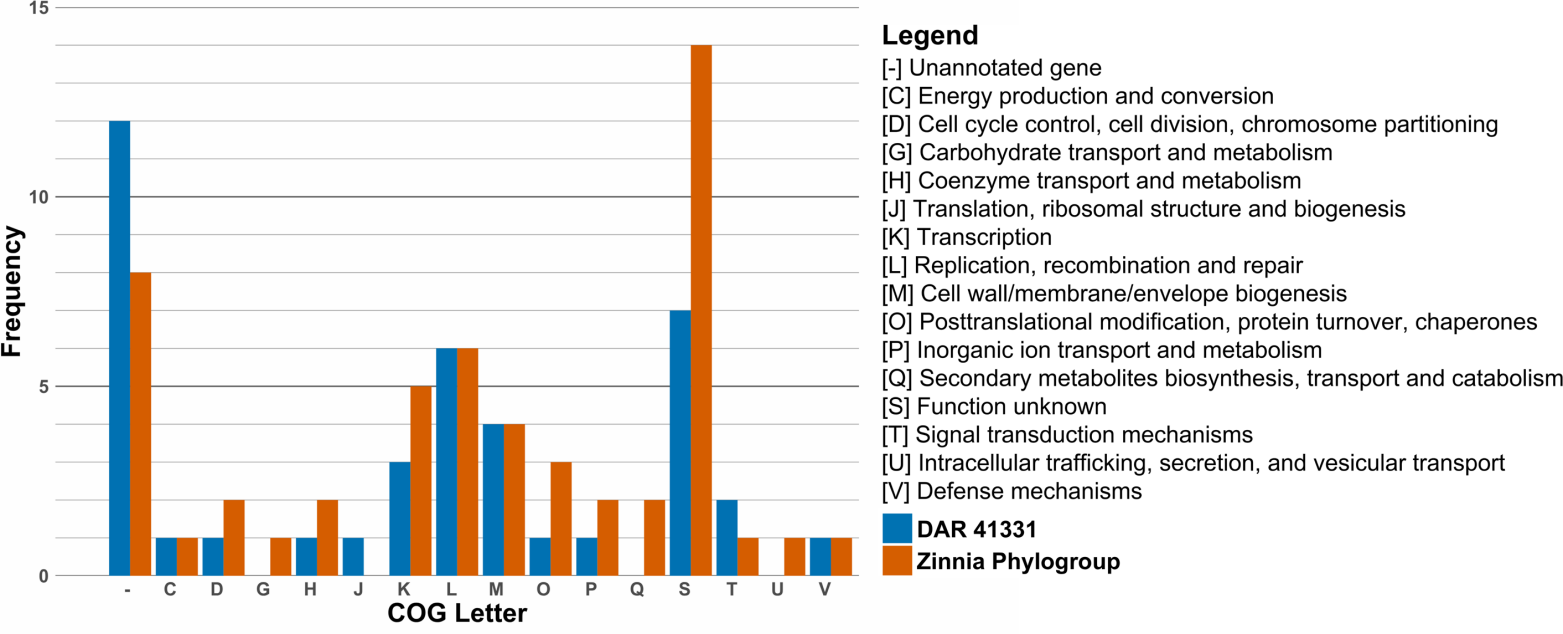
Genes unique to the zinnia phylogroup and DAR 41331, grouped by functionality in COG categories. Frequency shows how many genes belong to each category.

Further investigations sought to determine what genetic content is unique to the zinnia phylogroup (DAR 41374, DAR 82727, DAR 82752, DAR 82756 and NCPPB 2439) and DAR 41331 compared to the other members of the ‘*X. cannabis*’ species. The resulting sets of unique genes for the zinnia phylogroup and DAR 41331 are visualized in Fig. 5. EggNOG Mapper outputs are available in Tables S5 and S6, with the corresponding gene coordinates for each genome detailed in Tables S7 and S8.

Our pangenome analysis and filtering performed using Roary and Scoary initially identified 143 unique genes in the zinnia phylogroup. EggNOG Mapper was only able to annotate 97 of these genes using Clusters of Orthologous Groups (COG) categories. To confirm the uniqueness of these genes within the species, these genes were then compared to all other members of the ‘*X. cannabis*’ species using blastp which filtered the list down to 50 genes unique to the zinnia phylogroup for the final analysis. Similarly, DAR 41331 had 150 unique genes initially identified by Scoary, with 107 annotated by EggNOG mapper. After the same blastp cross-referencing, 40 genes unique to DAR 41331 were retained.

The number of unique genes is not exactly equal to the frequency of each category, as some genes are classified as more than one COG. We identified many proteins classified as [-] and [S], both of which represent proteins with unknown functions. Proteins in category [S] show matches to a protein family or domain of unknown function in EggNOG Mapper searches, whereas proteins in category [-] do not match any known proteins or protein domains. 

The most prevalent COG category of known function in DAR 41331 was [L] (replication, recombination and repair) with six genes. This included two reverse transcriptases, two helicase nucleases, a phage integrase and a DNA polymerase III. Of particular interest, DAR 41331 possessed a histone-like nucleoid structuring protein labelled as a virulence regulator, a TonB-dependent receptor associated with nutrient acquisition and two enzymes associated with polysaccharide biosynthesis. A variety of other enzymes were also detected, including cytidylyltransferase, methyltransferase, maltose O-acetyltransferase and adenylate and guanylate cyclase catalytic domain.

For the zinnia phylogroup, the most prevalent COG category of known function was also [L]. It included a protein kinase, a Mu transposase, a helicase domain and three ATPases. Other genes associated with genetic regulation that were detected include sigma 70 initiation factor *rpoD* and TetR family transcriptional regulator. They also possess two TonB-dependent receptors, which are different from each other and to the one found in DAR 41331.

Further investigation of the genes unique to the zinnia phylogroup revealed that they possess the T3Es *xopAU, xopD*, *xopK*, *xopL* and *xopR*. All but *xopAU* were included in the database used for our blastx analysis (Fig. 4) but were not identified as significant matches. Further inspection showed that the four Xop genes were detected in our blast analysis but exhibited sequence identity matches less than 56.2% when compared to our reference sequences. Combined, the EggNOG Mapper and blast data indicate that these five isolates contain seven to nine T3Es each.

Another notable feature of the zinnia phylogroup is the presence of *sugE*, which encodes a small multidrug resistance (SMR) pump. We also identified a family C39 peptidase, known for its processing and translocations of bacteriocins. Four bacteriophage genes were detected, a phage anti-repressor protein, two lambda head decoration proteins and the Mu transposase mentioned above (*tniA*). Lastly, this analysis identified a range of enzymes including nitroreductase, peptidase, sulfotransferase, methyltransferase and glycosyl transferase.

**Fig. 6.**
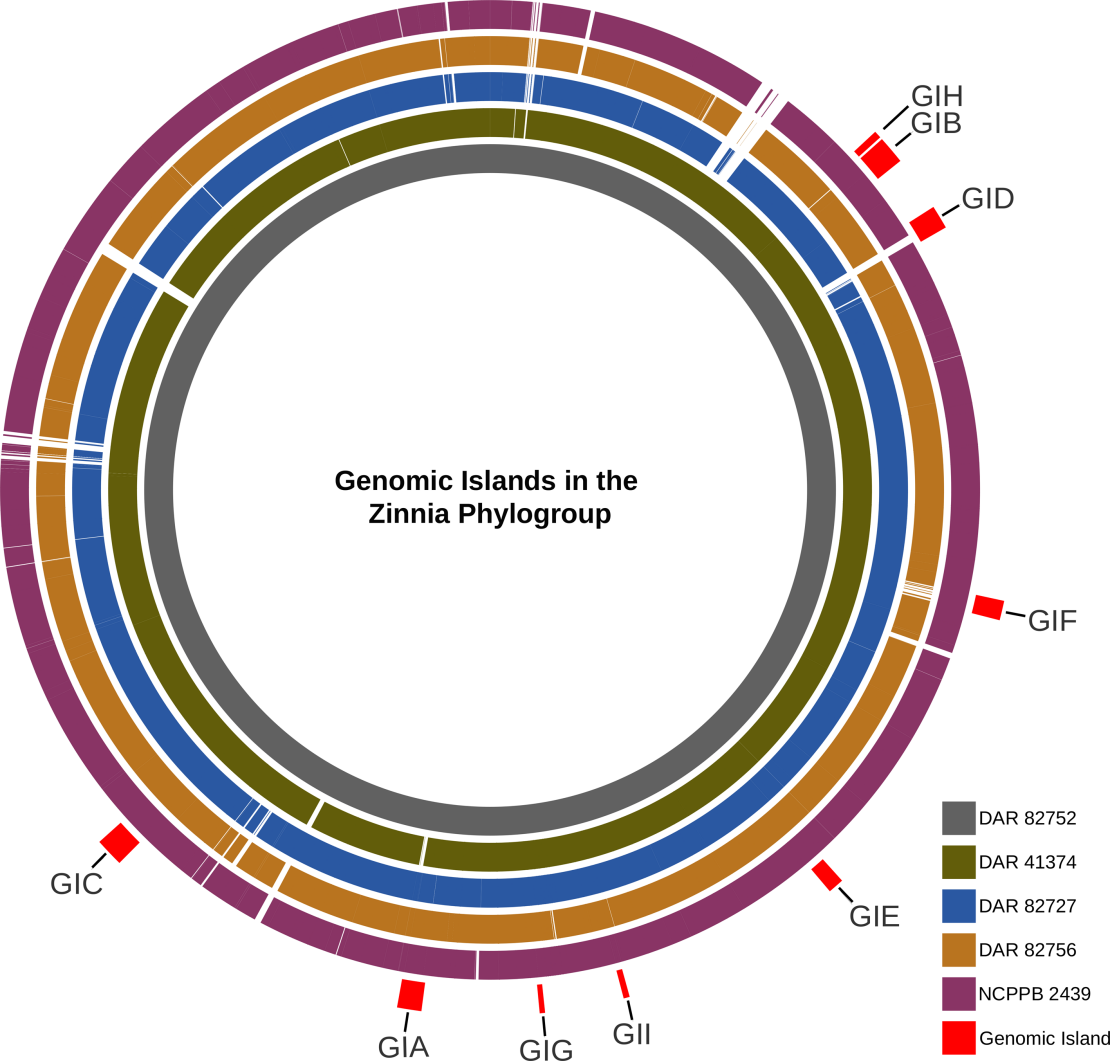
BRICK diagram showing the presence of GI in each of our isolates using DAR 82752 as the reference. From innermost to outermost, the genomes are ordered as DAR 82752, DAR 41374, DAR 82727, DAR 82756 and NCPPB 2439. GIs are labelled on the outermost red blocks.

After examining the unique genetic content of the zinnia phylogroup, we investigated the presence of GIs in their genomes. Nine GIs (GIA– GII) were identified in at least three of the five isolates (Fig. 6). There were eight GIs that contained mobile elements (i.e. conjugative transfer proteins, integrase or transposase), four included tRNA or tmRNA genes, while three of them harboured virulence-associated factors. This suggests the presence of multiple pathogenicity islands (PIs) within this phylogroup. Three of the four containing tRNA/tmRNA were found to be inserted at the 3′-end of the sequence, typical of GI [86,87]. GI ranged from 8,300 to 49,500 bp in length containing between 9 and 46 genes. The GC content ranged from 56.1–61.7 mol%, while the average whole genome GC content was 65.4–65.7 mol%. The GI was similar in size and composition between isolates, but GIA in the NCPPB 2439 assembly was cut short, most likely due to it being positioned at the end of a contig. Table S4 contains the GC percentage, gene content and coordinates of the GI in each isolate. Alignment revealed that the GIs exhibited genetic variability across different regions in each isolate. It showed that while most GI remained largely intact, some lacked or possessed genes not found in others.

The GIs were not found in any other members of the ‘*X. cannabis*’ species besides our other isolate, DAR 41331, which only possessed GIC, the largest GI with the most virulence-associated content. However, while this GI was highly conserved in other isolates, ~25% of it was missing in DAR 41331 and contained regions of genetic variability. None of the GIs were found in any of the *Xanthomonas* type strains besides GIF which was detected in *Xanthomonas vesicatoria*. Using blast, we screened for the nine GIs in both the NCBI database and our own collection of over 900 *Xanthomonas* isolates gathered through routine biosecurity surveillance. The only GI detected in our database was GIF, which was identified in four *X. vesicatoria* isolates. In the NCBI database, GIA, GIB and GIH had no significant hits. Conversely, GIC, GID, GIG and GII yielded numerous high similarity matches with only 50–60% coverage. Applying a sequence length and similarity threshold ≥70% and an e-value less than 1e^−10^, GIE and GIF were detected in 30 and 25 isolates, respectively. Species containing GIE included *Xanthomonas arboricola, X. campestris*, *Xanthomonas citri*, *Xanthomonas euroxanthea*, *Xanthomonas fragariae*, *Xanthomonas hortorum*, *X*. *oryzae*, *Xanthomonas prunicola and Xanthomonas vasicola*. GIF was mainly detected in *X. campestris* pv. *campestris* but was also found in *Xanthomonas phaseoli* and *X. vesicatoria*.

Amongst the nine GIs, we identified a diverse collection of genes with different functions. These included mobile genetic elements such as transposons, transposases, integrases, site-specific integrases, recombinases and plasmid mobilization protein MobA/MobL. In addition, we found general-purpose genes like DNA repair genes (*uvrB* and *radC*) and biosynthesis-associated genes (*mdcH*, *mdcB*, *mdcG* and *mdcE*) which are involved in the synthesis of fatty acids from malonate. The tRNA genes that encode for Val, Asn, Ser, Met and Gln were detected. Metabolism-associated proteins were identified like lipases, serine protease and molybdopterin synthase/*moaE*/*moaD*/*moaE* and *panE* (vitamin B5 synthesis). There were also more miscellaneous genes like *dnaX* (polymerase subunit III), abortive infection family protein, toxin–antitoxin system, caspase (programmed cell death), outer membrane adhesion genes (*ompV*), many transcriptional regulators, pilus assembly genes, ATPase, reverse transcriptase and multidrug efflux SMR transporter (*emrE*). We also detected multiple phage-associated genes, including bacteriophage portal protein and AlpA family phage regulatory protein. In GIC, PHASTEST identified a contiguous cluster of six bacteriophage genes, comprising a head protein, a portal protein, two tail proteins, a hypothetical protein and DNA helicase. All GI contained a total of ~150 unannotated hypothetical proteins.

**Fig. 7.**
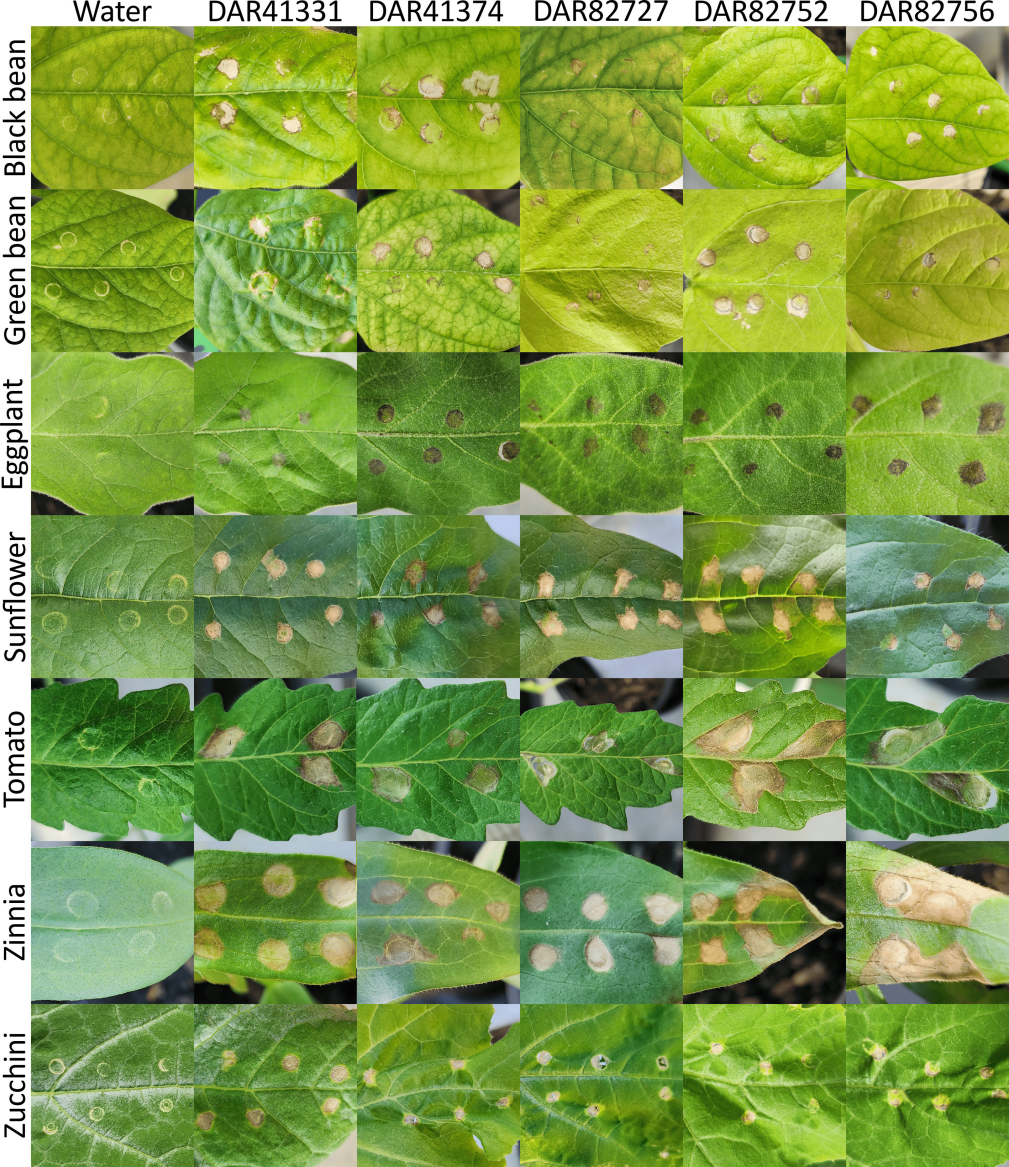
Symptoms caused by Australian ‘*X. cannabis’* isolates on various plant species compared to water-inoculated controls. Shown are leaves of black bean, green bean, eggplant, sunflower, tomato, zinnia and zucchini 7–14 days post-inoculation.

To evaluate the pathogenic potential of our five Australia ‘*X. cannabis*’ isolates, we conducted an HR assay using black bean, eggplant, green bean, tomato, sunflower, zinnia and zucchini plants. All inoculated plants, except the water controls, consistently developed symptoms characteristic of a *Xanthomonas* infection, achieving a 100% infection rate. Initial symptoms, observed ~48 h post-inoculation, appeared as sunken, translucent or brown spots localized around the infiltration site (Fig. 7). While the overall appearance of symptoms was broadly similar across plant species, some variations were noted, even within individual plant hosts. Over time, some leaf spots progressed to become necrotic, drying out and turning brown. Leaf spots on the eggplant leaves appeared to be a darker brown compared to that of other plant species. Black bean, green bean, sunflower, tomato and zinnia plants frequently developed angular leaf spots, characteristically bordered by the leaf veins. In some zinnia plants, these lesions extended to the leaf edge, leading to drying and curling. Similarly, some bacterial leaf spots on tomato plants became so dry and thin that they formed a hole. No single isolate appeared more virulent than the others, as all atypical symptoms were broadly observed across all tested isolates.

To confirm the identity of the causal agent, bacterial isolates were recovered from symptomatic plant tissue and analysed using MALDI-TOF MS. The resulting spectra of DAR 41331 consistently and unambiguously matched with itself with scores ranging from 2.5 to 2.6, well above the species-level identification threshold (≥2.0). The remaining four isolates (DAR 41374, DAR 82727, DAR 82752 and DAR 82756) also generated high-confidence identification scores (2.3 to 2.6), typically matching themselves as the closest hit. However, due to their extremely similar proteomic profiles, occasionally the highest match was to a different isolate within this cluster. In these cases, the correct isolate was still confidently identified as a top hit with a score≥2.3.

## Discussion

Here, we report confirmation of ‘*X. cannabis*’ in Australia as, due to recent proposed reclassifications, no previous studies have fully documented its presence on the continent. Prior to our investigation, NCPPB 2439 was the only isolate of the ‘*X. cannabis*’ species that had been collected in Australia. However, previous studies that included information about NCPPB 2439 either did not provide the country of origin or, due to the recent proposed reclassification of pv. *zinniae*, named the organism as *X. campestris* [39,40, 43, 88,90]. The reported presence of ‘*X. cannabis*’ in Australia is significant for our agricultural industries given its pathogenicity and broad host range.

The large potential host range of these isolates was confirmed in our plant HR trials. ‘*X. cannabis*’ has previously been shown to infect barley, multiple bean varieties, cannabis, capsicum, dahlia, geranium, Japanese fig, mulberry, tobacco, tomato and zinnia plants in laboratory conditions [17,41, 46, 90]. As such, we included a variety of plant hosts within the known host range as well as logical additions. Zinnia and zucchini were chosen for our HR trial to confirm our isolates could cause disease in their original host plants. The purple prince zinnia variety was chosen due to it being highly susceptible to pv. *zinniae* [45]. Sunflowers are both an ornamental flower and food crop and are in the *Asteraceae* family with zinnia, so they were added to the study. Tomato and capsicum have been shown to be susceptible to ‘*X. cannabis*’ [17,41] and so tomato and an additional solanaceous plant, eggplant, were chosen. Lastly, 8 out of 20 ‘*X. cannabis*’ isolates have been found on beans and many have been shown to cause disease under laboratory conditions [88,90], so both green and black beans were included.

Our results reflect that of other studies with the successful infection of tomato, zinnia and both green and black bean plants. However, it also expands the known host range of ‘*X. cannabis*’ to include eggplant, sunflower and zucchini plants. Past studies used pinprick inoculation, sprayed the plants with water or used a needleless syringe [17,41, 90]. We used a needleless syringe as it was the most promising in our pilot study and decreased the likelihood of cross-contamination. Symptoms appeared within 48 h, like in previous reports [17,41, 90]. The past syringe study described water-soaked lesions in capsicum, hypersensitivity reactions in tobacco and leaf yellowing and necrosis around the infiltration zone [17]. By contrast, in all plants, we observed necrosis around the injection site, leading to a translucent appearance and small amount of spreading. Additionally, black bean, green bean, sunflower, tomato and zinnia plants had angular leaf spots that spread down the leaf towards the apex but were restricted by the veins.

The re-isolation and identification of the ‘*X. cannabis*’ strains from plant hosts in the HR trial was successful, confirming their presence as the causal agent. However, the MALDI-TOF MS method was shown not to precisely differentiate four of the five isolates (DAR 41374, DAR 82727, DAR 82752 and DAR 82756). While DAR 41331 consistently matched its reference, the other four isolates occasionally produced slightly higher scores with each other than their own reference. This difficulty likely stems from their high genomic similarity, which results in insufficient proteomic differences for clear differentiation by MALDI-TOF MS, a challenge well documented in the literature for highly similar organisms [91,92]. Although precise discrimination between some of the isolates was not possible, the purpose of the MALDI-TOF to confirm that ‘*X. cannabis*’ was the causal agent was achieved. Further, stringent contamination control measures were employed throughout the study with no symptoms observed in the water-treated controls. These findings provide strong evidence that the bacterium responsible for the observed symptoms was successfully re-isolated and identified as ‘*X. cannabis*’.

Our results indicate that DAR 41331 lacks the genes necessary for T3SS and T3E, yet it performed very similarly to that of the other Australian ‘*X. cannabis*’ isolates in our HR trial. Our data also revealed that 12 of 15 other members of the ‘*X. cannabis*’ species also lack T3SS and T3E, despite many being known pathogens. Past studies have reported similar findings, showing that two ‘*X. cannabis*’ strains lacking Hrp T3SS and T3E still cause disease in multiple plant species [17]. This same study showed that despite lacking the Hrp T3SS, these strains possessed the Hrp virulence regulators, HrpG and HrpX. Our analysis has revealed that all publicly available members of ‘*X. cannabis*’ have both HrpG and HrpX. Of these 20 strains, only Nyagatare, CFBP7912 and the zinnia phylogroup possess T3SS and T3E. Given that ‘*X. cannabis*’ strains appear to exhibit similar virulence regardless of the presence of Hrp T3SS, this suggests that the T3SS is not the primary disease mechanism in ‘*X. cannabis*’. Jacobs *et al*. [17] showed that HrpX targets the promoter for two polygalacturonases, one putative aminopeptidase and one putative lysophospholipase that are found in nearly all *Xanthomonads* [17]. Further investigations should determine the role of these enzymes during plant colonization and measure their virulence in their absence. This will allow us to better understand the pathogenicity of the ‘*X. cannabis*’ as well as the disease mechanisms of the broader *Xanthomonas* genus, which would allow more effective management during outbreaks.

Our pangenome analysis revealed that both DAR 41331 and the zinnia phylogroup contain a variety of genes that may aid in virulence, survivability and adaption. Of particular interest was the detection of *sugE* in the zinnia phylogroup. This gene encodes an SMR efflux pump that confers resistance to a range of disinfectants, detergents and laboratory reagents. These include quaternary ammonium compounds, benzalkonium chloride, cetylpyridinium chloride, cetyltrimethylammonium bromide, ethidium bromide, SDS and tetraphenylphosphonium [93,94]. Future studies should investigate if the zinnia phylogroup exhibits increased resistance to these compounds and whether this provides any advantage against chemicals used in agricultural settings.

The discrepancy in the detection of Xop genes in our EggNOG Mapper and blastx results can be attributed to the differences in reference databases. EggNOG Mapper has access to a more comprehensive dataset, which includes multiple genetic variants of each gene across various organisms, including two group 2 *Xanthomonas* species. In contrast, our curated blast reference database, while specific, was inherently limited by the quality and quantity of our reference sequences. Creating an exhaustive database of virulence-associated factors for ‘*X. cannabis*’ or its close relatives is a significant challenge. Consequently, the more diverse sequence representations in EggNOG Mapper’s database appear to have enabled the detection of these more distant homologues that fell below our blast thresholds. These findings highlight the complementary strengths of these two approaches and demonstrate how using multiple analytical methods can provide a more complete picture of gene content in bacterial genomes.

Beyond virulence-associated factors, our analysis also revealed that nearly all ‘*X. cannabis*’ strains in this study possess the oxidoreductase and transcriptional regulator for cercosporin degradation, including the *zinniae* pathotype strain (NCPPB 2439). This finding is consistent with past studies that demonstrated that this strain consistently exhibited cercosporin degradation [47]. The fixed presence of these genes for cercosporin degradation across many ‘*X. cannabis*’ strains further validates the promising avenues for future research. This could include use in biological control strategies, such as engineering cercosporin-resistant crops or harnessing attenuated bacteria as biocontrol agents.

This study represents the first analysis of GIs in ‘*X. cannabis*’, identifying nine GIs across the zinnia phylogroup. Our findings reveal that these GIs share characteristics previously observed in other *Xanthomonas* species, such as varied GC content, the presence of mobile elements, flanking tRNAs and typical size ranges [95,97], as well as an abundance of virulence factors. Their unique genomic content identified by pangenome analysis combined with the identification of genomic regions acquired through horizontal gene tranfer (HGT) highlights the uniqueness of the zinnia phylogroup within the species.

A 2022 study of 81 genomes from four group 2 *Xanthomonas* species found that PIs were generally conserved within strains of the same pathovar or subspecies [95]. Our findings align with this observation, as the identified GIs were absent in other ‘*X. cannabis*’ isolates, with the notable exception of the partial GI in DAR 41331. Furthermore, the genomic content of the GIs analysed in our study mirrors that reported in the aforementioned research, containing integrases, pilus genes, conjugative elements, T4SS, transposases, prophage regions and a significant number of hypothetical proteins [95].

Previous investigations of GIs in other group 2 *Xanthomonas* species have identified large GIs that confer resistance to copper, likely a consequence of the widespread use of copper bactericides in agriculture [98,99]. Similarly, our GI analysis identified the SMR drug pump, EmrE, within all zinnia phylogroup strains except NCPPB 2439. EmrE utilizes the proton gradient across the inner membrane of bacterial cells to translocate toxic cationic compounds out of the cell [100,101]. This confers resistance to antiseptics like benzalkonium and acriflavine, antibiotics like streptomycin and tobramycin, and the herbicide methyl viologen [102,105]. The presence of both EmrE and SugE SMR efflux pumps in these isolates warrants further studies examining their resistance to antibiotics, disinfectants and agriculturally relevant compounds.

Of the nine GIs we detected, three were not observed in any isolates in the NCBI database or within the ‘*X. cannabis*’ genus. This suggests a recent acquisition, most likely through HGT, from a source that is not currently represented in the NCBI database. Future investigations should determine whether this acquisition has impacted their virulence, host range or adaptability.

By contrast, four GIs showed high similarity with regions of publicly available genomes but were missing approximately half of their genomic content. Two other GIs had high similarity and length matches across various group 2 *Xanthomonas* species. This could indicate that a distant common ancestor acquired these six GIs prior to speciation and they were uniquely conserved within members of ‘*X. cannabis*’. It is also possible that the four GIs with missing content were merely separated through recombination, causing them to appear incomplete in the blast search. Future studies could analyse the metadata of all isolates containing these GIs to investigate correlations between their time and place of collection and presence of these GIs. These analyses would help determine if these GIs were acquired post-speciation. Past studies have successfully used genomic analyses such as molecular clock analysis, phylogenetics and Bayesian inference to study the impacts of HGT on evolution [106,109]. This approach would allow us to better understand how HGT has impacted the genetic diversity, evolution and pathology of the *Xanthomonas* genus.

The absence of these GIs in other pathogenic ‘*X. cannabis*’ isolates suggests that they do not contain the primary virulence mechanisms of the species. However, the acquisition of unique GIs highlights that established pathogen populations can represent a latent and evolving threat. If these pathogens are detected during future outbreak surveillance and sequenced, it would provide valuable insights into how they have evolved in response to agricultural practices and environmental pressures.

Our findings enhance our understanding of ‘*X. cannabis*’ by presenting all sequenced isolates with their respective metadata and virulence profile for the first time. Further, the confirmation of ‘*X. cannabis*’ in Australia clarifies the geographic distribution of this species. However, despite clear evidence presented in multiple publications, ‘*X. cannabis*’ is not currently classified as a valid species under the International Code of Nomenclature of Prokaryotes. To address this and provide an effective publication for valid classification, we have included a descriptive protologue for ‘*X. cannabis*’ adapted from publications that have described ‘*X. cannabis*’ previously [17,38, 41]. A valid description will aid in future identification of ‘*X. cannabis*’ as continuous surveillance and characterization of emerging strains is crucial for effective biosecurity management and robust disease control strategies. Future ‘*X. cannabis*’ research should investigate its primary disease mechanism, the extent of its host range, drug resistance and cercosporin biocontrol strategies.

## Description of *Xanthomonas cannabis* sp. nov.

*Xanthomonas cannabis* sp. nov. (*can.na.bis*. L. fem. n. *cannabis*, of cannabis, referring to *Cannabis sativa*, the host from which the bacterium was first isolated).

As described in Severin [38], cells are rod-shaped, rounded at the ends and Gram-negative and form capsules on glucose-containing substrates but not NA medium. Colonies on NA medium appear after 48 h and are slightly convex, glossy and yellowish and have a smooth margin. The yellow pigment is an alcohol carotenoid of the ‘*Xanthomonas*’ type. Forms a yellow ring on sugar-containing media. Milk is peptonized, and starch and aesculin are hydrolysed, whereas with arbutin, only weak hydrolysis is detectable, and casein is not reactive. Proteolytic activities are present. Nitrates are not reduced. Hydrogen sulphide and ammonia formation are detectable. Voges–Proskauer and methyl red tests are negative. Kovac’s oxidase test and the purple lactose test are also negative. Growth is good in Fermi’s synthetic medium. The majority of isolates grow well in Uschinsky’s solution, but not in Cohn’s solution. From arabinose (9/12), xylose, glucose, levulose, galactose, mannose (11/12), sucrose, maltose, raffinose, lactose, glycerol, mannitol (11/12), sorbose and cellobiose, acid is formed, but no gas. Salicin is not degraded. Water-soaked lesions have been observed in *P. vulgaris*, *Hordeum vulgare*, *Dahlia pinnata*, *S. lycopersicum*, *Pelargonium*, *Morus* sp., *Ficus erecta*, *Cannabis sativa*, *Capsicum annuum* and *Zinnia* sp. [17,41, 46, 90]. *Pelargonium zonale*, *Glycine hispida* and *Nicotiana tabacum* show a typical hypersensitivity reaction [17,38]. *Cucumis sativus* are not affected [38]. Leaf spotting was observed in *H. vulgare* L. Morex under experimental conditions [38]. Brown dry leaf spots have been observed in *S. lycopersicum*, *S. melongena*, *C. pepo*, *H. annuus*, *Zinnia* spp. and *P. vulgaris*.

The species type strain is NCPPB 2877^T^=LMG 9042^T^=ICMP 6570^T^. The type strain genome assembly (GCF_000802365.1) has a G+C content of 65.8 mol% and a length of 4.76 Mbp [17]. The type strain genome sequence has been independently confirmed by sequencing of *gyrB* [41]. The type strain was isolated in 1974 from a leaf spot on *C. sativa* in Lovrin, Romania.

Accession numbers for raw reads available in the GenBank database

**Table:** 

Isolate **code**	Read **accession**	Sequencing type
DAR 82756	SRR25298666	Illumina
DAR 82752	SRR25298667	Illumina
DAR 82727	SRR25298668	Illumina
DAR 41374	SRR25298669	Illumina
DAR 41331	SRR25298670	Illumina
DAR 82756	SRR25298671	Oxford Nanopore
DAR 82752	SRR25298672	Oxford Nanopore
DAR 82727	SRR25298673	Oxford Nanopore
DAR 41374	SRR25298674	Oxford Nanopore
DAR 41331	SRR25298675	Oxford Nanopore

Accession numbers for assembled genomes available in the GenBank database

**Table:** 

Isolate **code**	Biosample **accession**	Genome **accession**
DAR 41331	SAMN35449586	CP136580
DAR 41374	SAMN35449587	CP136579
DAR 82727	SAMN35449588	CP136578
DAR 82752	SAMN35449589	CP136577
DAR 82756	SAMN35449590	CP136576

## Supplementary material

10.1099/mgen.0.001588 Uncited Supplementary Material 1.

## Abbreviations

ANI: average nucleotide identity
COG: Clusters of Orthologous Groups
EPS: exopolysaccharide
GI: genomic island
HGT: horizontal gene tranfer
HR: hypersensitivity response
LPS: lipopolysaccharide
NA: nutrient agar
NCBI: National Center for Biotechnology Information
NSW: New South Wales
PI: pathogenicity island
PIP: plant-induced promoter
QLD: Queensland
SMR: small multidrug resistance
T3E: type 3 effector
T1SS: type 1 secretion system
T2SS: type 2 secretion system
T3SS: type 3 secretion system
T4SS: type 4 secretor system
T5SS: type 5 secretion system
T6SS: type 6 secretor system
